# Combinatorial CRISPR Interference Library for Enhancing 2,3-BDO Production and Elucidating Key Genes in Cyanobacteria

**DOI:** 10.3389/fbioe.2022.913820

**Published:** 2022-06-21

**Authors:** Hung Li, Nam Ngoc Pham, Claire R. Shen, Chin-Wei Chang, Yi Tu, Yi-Hao Chang, Jui Tu, Mai Thanh Thi Nguyen, Yu-Chen Hu

**Affiliations:** ^1^ Department of Chemical Engineering, National Tsing Hua University, Hsinchu, Taiwan; ^2^ Department of Life Science, National Taiwan University, Taipei, Taiwan; ^3^ Department of Chemical Engineering, National Taiwan University, Taipei, Taiwan; ^4^ Faculty of Chemistry, University of Science, Vietnam National University Ho Chi Minh City, Ho Chi Minh City, Vietnam; ^5^ Frontier Research Center on Fundamental and Applied Sciences of Matters, National Tsing Hua University, Hsinchu, Taiwan

**Keywords:** cyanobacteria, PCC7942, CRISPR, CRISPRi library, combinatorial metabolic engineering

## Abstract

Cyanobacteria can convert CO_2_ to chemicals such as 2,3-butanediol (2,3-BDO), rendering them promising for renewable production and carbon neutralization, but their applications are limited by low titers. To enhance cyanobacterial 2,3-BDO production, we developed a combinatorial CRISPR interference (CRISPRi) library strategy. We integrated the 2,3-BDO pathway genes and a CRISPRi library into the cyanobacterium PCC7942 using the orthogonal CRISPR system to overexpress pathway genes and attenuate genes that inhibit 2,3-BDO formation. The combinatorial CRISPRi library strategy allowed us to inhibit *fbp*, *pdh*, *ppc,* and *sps* (which catalyzes the synthesis of fructose-6-phosphate, acetyl-coenzyme A, oxaloacetate, and sucrose, respectively) at different levels, thereby allowing for rapid screening of a strain that enhances 2,3-BDO production by almost 2-fold to 1583.8 mg/L. Coupled with a statistical model, we elucidated that differentially inhibiting all the four genes enhances 2,3-BDO synthesis to varying degrees. *fbp* and *pdh* suppression exerted more profound effects on 2,3-BDO production than *ppc* and *sps* suppression, and these four genes can be repressed simultaneously without mutual interference. The CRISPRi library approach paves a new avenue to combinatorial metabolic engineering of cyanobacteria.

## Introduction

Bio-based production of chemicals from renewable resources draws increasing attention due to growing concerns on environmental sustainability and global warming (Chae et al., 2020). Cyanobacteria are photoautotrophic prokaryotes capable of converting CO_2_ to organic compounds *via* photosynthesis. These useful traits have led to genetic manipulation of cyanobacteria including *Synechococcus elongatus* PCC7942 and *Synechocystis sp* PCC6803 for production of industrially relevant chemicals such as 2,3-butanediol [2,3-BDO), ethanol, acetone, and isopropanol (for review see (Zhou et al., 2016; Stephens et al., 2021)]. However, the product titers are usually much lower than those from other microbial hosts (Zhao et al., 2021). In the case of 2,3-BDO, which is used in the food, fine chemical, cosmetics, and pharmaceutical industries (Liao et al., 2016), metabolically engineered PCC6803 produces 0.43 g/L of 2,3-BDO (Savakis et al., 2013), while PCC7942 confers 2,3-BDO titers from 0.496 g/L to 2.38 g/L (Oliver et al., 2013; Oliver et al., 2014). More recently, the carbon metabolism of PCC7942 was engineered to improve glucose utilization, enhance CO_2_ fixation, and increase the 2,3-BDO titer to 12.6 g/L (Kanno et al., 2017). Yet, this titer is still lower than those using other metabolically engineered organisms (Kim et al., 2013; Li et al., 2015). Production efficiency poses a challenge in developing cyanobacteria for chemical production (Shabestary et al., 2018; Liu et al., 2022).

Synthetic biology and metabolic engineering have converged to allow the construction of cell factories for the synthesis of high-value-added biochemicals (Zhao et al., 2021). In recent years, various strategies have been developed to modulate the expression of pathway genes and regulate metabolic networks (Zhao et al., 2021), such as MAGE (multiplex automated genome engineering), CoS-MAGE (co-selection MAGE) and COMPACTER (customized optimization of metabolic pathways by combinatorial transcriptional engineering). Apart from these methods, the RNA-guided genome editing tool CRISPR has been used to insert or delete pathway genes for microbial metabolic engineering (Chung et al., 2017; Pham et al., 2020; Zhao et al., 2021). CRISPR requires expression of Cas nuclease such as Cas9 and chimeric single-guide RNA (sgRNA), comprising the spacer and scaffold motif (Jinek et al., 2012). sgRNA coordinates with Cas9 to recognize the protospacer-adjacent motif (PAM) on the target DNA, with the guide by the spacer sequence. By changing the spacer sequence, one may harness the Cas9/sgRNA complex to specifically recognize the target gene for programmable gene editing (Hsu et al., 2019; Doudna, 2020). We have previously shown that CRISPR improves gene integration into cyanobacterium PCC7942 with a shorter homology arm (Li et al., 2016), which demonstrates the potential of CRISPR for genome engineering of PCC7942.

CRISPR was also repurposed for CRISPR interference (CRISPRi) by using a catalytically inactive SpCas9 (SpdCas9), which orchestrates with sgRNA to inhibit target gene transcription (Qi et al., 2013; Behler et al., 2018; Hsu et al., 2020). SpdCas9-based CRISPRi can repress endogenous and heterologous gene expression in PCC6803 (Yao et al., 2016) and PCC7942 (Huang et al., 2016) in a reversible and titratable manner (Gordon et al., 2016; Yao et al., 2016). In PCC7942, integration of the SpdCas9-expressing cassette into the neutral site I (NSI) and sgRNA-expressing cassette into the neutral site II (NSII) enables repression of three endogenous genes in competing pathways at efficiencies up to 95%, resulting in a ≈12.5-fold enhancement of succinate production (Huang et al., 2016). However, CRISPR and CRISPRi are yet to be coupled to improve bio-derived product synthesis in cyanobacteria.

To further enhance the cyanobacterial production of 2,3-BDO, here, we first explored orthogonal CRISPR systems to integrate 2,3-BDO pathway genes and the SpdCas9-based CRISPRi system into different NS sites in PCC7942 to simultaneously overexpress pathway genes and suppress potential genes that inhibit 2,3-BDO formation. We next developed a CRISPRi library approach to inhibit *fbp*, *pdh*, *ppc,* and *sps* at different levels in a combinatorial way. The CRISPRi library approach opens a new avenue to combinatorial metabolic engineering of cyanobacteria.

## Results

### Assessment of Orthogonal CRISPR Systems for Gene Integration Into PCC7942

**FIGURE 1:**
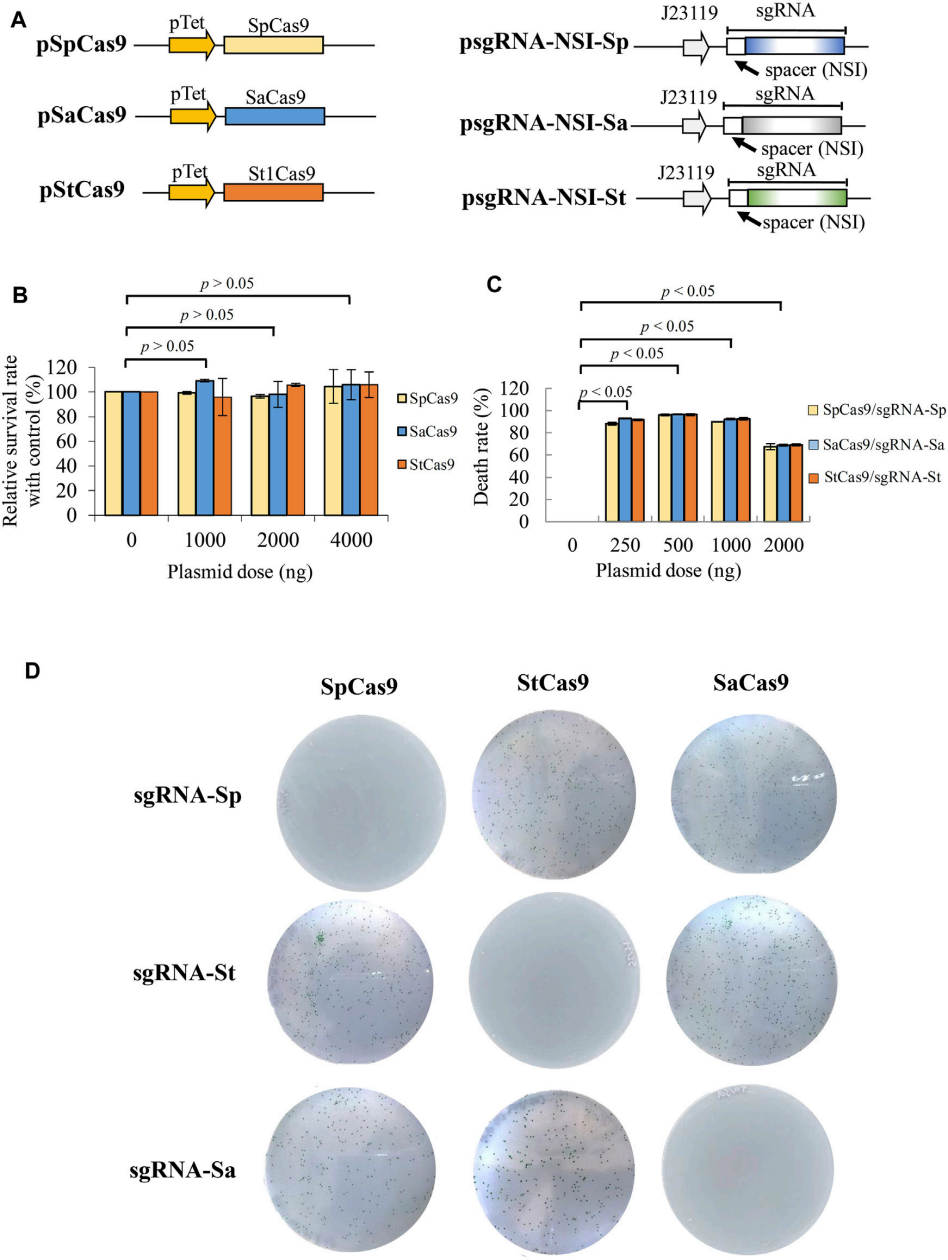
Validation of orthogonal CRISPR systems for gene editing in PCC7942. **(A)** Plasmids encoding SpCas9, SaCas9, and St1Cas9 as well as plasmids encoding their corresponding sgRNA. Each sgRNA comprised identical spacer targeting NSI site but different scaffold backbone. sgRNA was driven by J23119 promoter. **(B)** Survival rate relative to the control. Cas9-expressing plasmid was transformed alone at different doses (1,000–4,000 ng). Nontransformed cells (0 ng) served as the control. Survival rate was assessed by (CFU/CFUcontrol) × 100%. **(C)** Death rate induced by different cognate Cas9/sgRNA pairs. Cas9-expressing plasmid was co-transformed with the corresponding sgRNA-expressing plasmid at different doses, and the death rate was assessed using nontransformed cells (0 ng) as the control. The death rate was calculated by (1-CFU/CFUcontrol) × 100%. **(D)** Orthogonality test for three Cas9 variants. The Cas9-and sgRNA-expressing plasmids were co-transformed into PCC7942 and streaked to plates. The plates were observed at day 7–9. The data represent the mean ± SD of three independent culture experiments and were analyzed by one-way ANOVA. *p* < 0.05 was considered statistically significant.

The CRISPR system based on SpCas9 (Cas9 from *Streptococcus pyogenes*) can induce PCC7942 death and was harnessed for programmable gene integration into PCC7942 (Li et al., 2016). There are other Cas9 orthologs derived from different microorganisms such as *Streptococcus pyogenes* (SpCas9) (Cong et al., 2013; Mali et al., 2013), *Staphylococcus aureus* (SaCas9) (Ran et al., 2015), and *Streptococcus thermophilus* (St1Cas9) (Kleinstiver et al., 2015). These Cas9 variants can mediate genome editing with higher specificity and are orthogonal to one another in *E. coli* (Sung et al., 2019). However, whether they can mediate gene integration into PCC7942 is yet to be explored. To integrate the CRISPRi library into the cyanobacterial genome using CRISPR, while avoiding mutual interference, we evaluated SpCas9, SaCas9, and St1Cas9 and used pSpCas9, pSaCas9, and pSt1Cas9 to express these three proteins (Figure 1A). Because SpCas9, SaCas9, and St1Cas9 recognize different PAM sequences (NGG, NGGRRT, and NNAGAAW, respectively) and require different sgRNA handle regions, we also constructed plasmids (e.g. psgRNA-NSI-Sp) expressing their corresponding sgRNA handle regions and identical spacers to target the non-essential NSI site (Figure 1A).

We first transformed the Cas9-expressing plasmid alone at different doses and observed no apparent cell death when compared with cells without the Cas9-expressing plasmid (Figure 1B), verifying that all the three Cas9 proteins barely induced cytotoxicity in PCC7942. We next co-transformed the three cognate plasmid pairs and observed >90% cell death rate (Figure 1C) at 250∼1,000 ng, indicating that all the three CRISPR/Cas9 systems induced double strand break (DSB) and cell death at high frequencies. Importantly, SpCas9, SaCas9, and St1Cas9 systems were orthogonal to each other in PCC7942, as judged from complete cell death only when Cas9 was paired with its cognate sgRNA (e.g. SaCas9/sgRNA-Sa, Figure 1D). These data demonstrated the feasibility of using SaCas9 and St1Cas9 systems to integrate the SpdCas9-based CRISPRi system or vice versa.

### Genome Engineering and Generation of 2,3-BDO-Producing PCC7942 Using SaCas9

**FIGURE 2:**
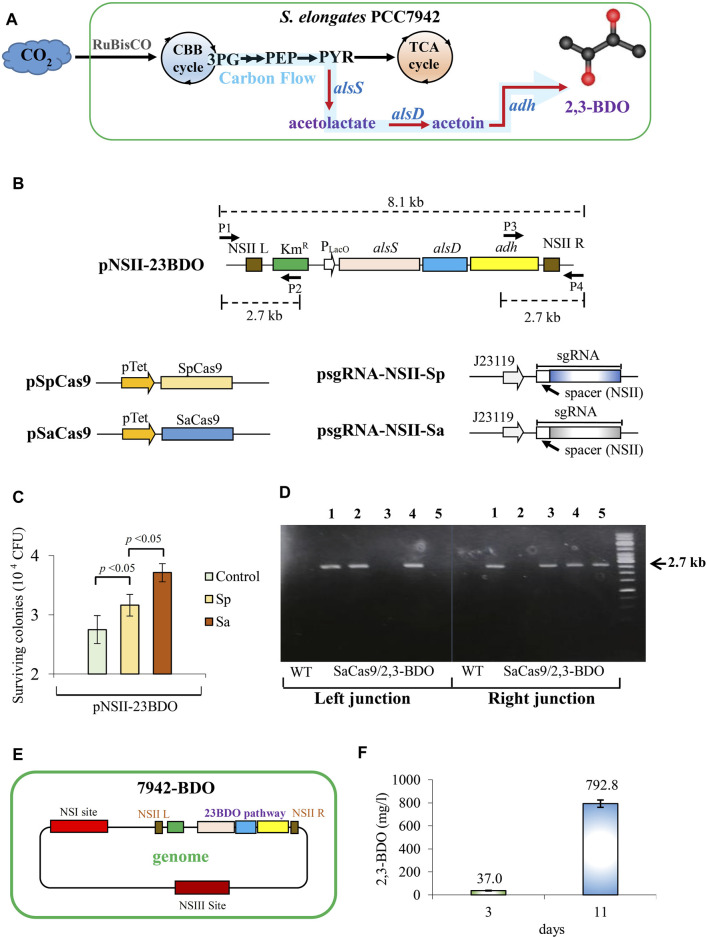
Generation of 2,3-BDO-producing PCC7942 using SaCas9. **(A)** Pathway leading to 2,3-BDO production. CBB, Calvin–Benson–Bassham cycle; 3 PG, 3-phosphoglycerate; PEP, phosphoenolpyruvate; PYR, pyruvate. **(B)** Plasmids encoding 2,3-BDO pathway genes driven by repressor-less LacO promoter (pNSII-23BDO) and sgRNA plasmids corresponding to SpCas9 (psgRNA-NSII-Sp) and SaCas9 (psgRNA-NSII-Sa). sgRNA spacer was designed to target the NSII site. NRII L and NSII R are the homology arms for the NSII site. Km^R^, kanamycin resistance gene. P1 to P4 are primers used for PCR check. **(C)** CFU after co-transformation of 2000 ng pNSII-23BDO, 500 ng of pSpCas9/pSgRNA-NSII-Sp (Sp group), or pSaCas9/pSgRNA-NSII-Sa (Sa group) and Km selection. The control was transformed with only 2000 ng pNSII-23BDO. **(D)** PCR analysis of five colonies from the Sa group using two primer pairs (P1/P2 and P3/P4) targeting the left and right junctions at the integration site. The expected PCR amplicon size is 2.7 kb. **(E)** Illustration of the 7942-BDO strain, which was picked after Km selection and re-streaking of colony 4 as shown in **(D)**. **(F)** 2,3-BDO titer after shake flask culture of 7942-BDO strain for 11 days. The quantitative data represent the mean ± SD of at least three independent culture experiments and were analyzed using Student’s *t*-test.

PCC7942 does not naturally produce 2,3-BDO. Nonetheless, 2,3-BDO can be synthesized with low cytotoxicity in PCC7942 if an artificial pathway comprising heterologous *alsS*, *alsD*, and *adh* (Oliver et al., 2013; 2014) is introduced to rewire the carbon flux from pyruvate (PYR) to 2,3-BDO (Figure 2A). To generate a 2,3-BDO-producing strain, we constructed pNSII-23BDO to encode heterologous *alsS*, *alsD*, and *adh* and homology arms targeting the NSII site (Figure 2B). Since the St1Cas9 PAM sequence was not found near the NSII site, we only constructed sgRNA plasmids corresponding to SpCas9 (psgRNA-NSII-Sp) and SaCas9 (psgRNA-NSII-Sa) (Figure 2B). We co-transformed pNSII-23BDO with pSpCas9/pSgRNA-NSII-Sp (Sp group) or pSaCas9/pSgRNA-NSII-Sa (Sa group) into PCC7942. As a control that represented the traditional transformation method, the cells were transformed with only pNSII-23BDO.

After selection and segregation, the Sa group conferred significantly higher colony-forming units (CFU) than the Sp and control groups (Figure 2C). Therefore, we randomly picked five colonies from the Sa group for PCR analyses using two primer pairs targeting the left and right junctions at the integration site (Figure 2B). The data confirmed successful integration in colonies 1 and 4 (Figure 2D). After re-streaking of colony 4 and culture in shake flasks, the final engineered strain (designated as 7942-BDO, Figure 2E) produced 792.8 mg/L of 2,3-BDO in 11 days (Figure 2F).

### Enhancing 2,3-BDO Production by CRISPRi Library Screening

**FIGURE 3:**
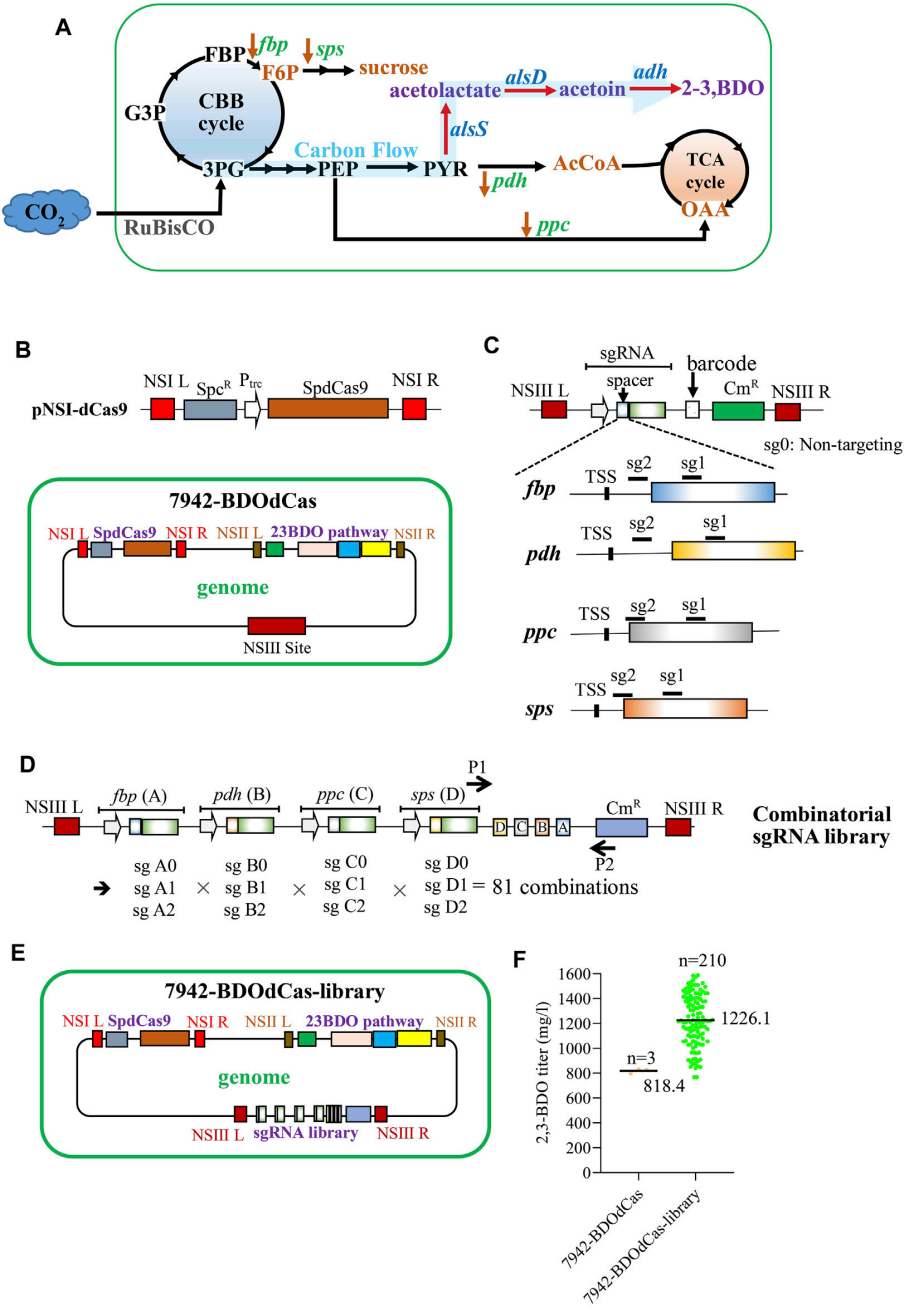
Development of the CRISPRi library for enhanced 2,3-BDO production. **(A)** Pathways leading to the synthesis of desired 2,3-BDO and undesired metabolites. AcCoA, acetyl-coenzyme A; OAA, oxaloacetate; F6P, fructose-6-phosphate; FBP, fructose-1,6-bisphosphate; *fbp*, fructose bisphosphatase; *pdh*, pyruvate dehydrogenase; *ppc*, phosphoenolpyruvate carboxylase; and *sps*, sucrose phosphate synthase. **(B)** Plasmid encoding SpdCas9 and the engineered 7942-BDOdCas strain. 7942-BDOdCas strain was engineered using SaCas9 to integrate SpdCas9 into NSI and 2,3-BDO pathway genes into the NSII site. **(C)** Plasmids encoding the sgRNA and unique barcode. The sgRNA spacer was designed to target the protospacer downstream the transcription start site (TSS) of *fbp*, *pdh*, *ppc*, or *sps* (sg1 or sg2) with the highest targeting scores. We also designed a plasmid encoding the scrambled sgRNA comprising a nontargeting spacer (sg0). **(D)** Illustration of the combinatorial sgRNA library with 81 combinations. Each sgRNA was labeled with a barcode (A: *fbp*; B: *pdh*; C: *ppc*; and D: *sps*; Supplementary Table S1) and was expressed from the same J23119 promoter. Each gene has three possible suppression levels (0: none; 1, weak; and 2: strong); thus, there are 3^4^ (=81) possible combinations. The combinatorial sgRNA library contained the homology arms for NSIII sites (NSIII L and NSIII R) The sequences were confirmed by PCR analyses of the unique barcode using primers P1/P2. **(E)** 7942-BDOdCas-library which was constructed by integrating the sgRNA library into the NSIII site of 7942-BDOdCas using SaCas9. **(F)** 2,3-BDO titer from 7942-BDOdCas-library. After antibiotic selection, 210 colonies were picked from 7942-BDOdCas-library and cultured in shaker flasks for 11 days for extracellular 2,3-BDO analyses using GC-BID. The quantitative data represent the mean ± SD of at least three independent culture experiments and were statistically analyzed by one-way ANOVA.

As shown in Figure 3A (Angermayr et al., 2015; Broddrick et al., 2016; Liu et al., 2022), in cyanobacteria, the Calvin–Benson–Bassham (CBB) cycle converts CO_2_ to 3-phosphoglycerate (3 PG), which can exit the cycle toward the synthesis of phosphoenolpyruvate (PEP) and pyruvate (PYR). PYR can be converted to acetyl-coenzyme A (AcCoA) by *pdh*-encoding pyruvate dehydrogenase. AcCoA reacts with oxaloacetate (OAA) to enter the noncanonical TCA cycle. Meanwhile, PEP can be converted to OAA by *ppc*-encoding phosphoenolpyruvate carboxylase. Conversely, in the CBB cycle, 3 PG continues to produce fructose-1,6-bisphosphate (FBP), which is converted to fructose-6-phosphate (F6P) by *fbp*-encoding fructose bisphosphatase. F6P can exit the CBB cycle and be converted to sucrose by *sps*-encoding sucrose phosphate synthase. Since the TCA cycle and FBP/sucrose synthesis compete with 2,3-BDO production, we hypothesized that inhibiting *fbp*, *pdh*, *ppc*, and *sps* by CRISPRi can attenuate the carbon partition toward TCA and F6P/sucrose pathways, thereby enhancing 2,3-BDO synthesis.

For CRISPRi integration, we first integrated the SpdCas9 expression cassette into the NSI site of the 7942-BDO strain with the SaCas9 system to avoid mutual interference, yielding the 7942-BDOdCas strain (Figure 3B). 7942-BDOdCas produced similar levels of BDO (818.4 mg/L) as compared with 7942-BDO (792.8 mg/L, Supplementary Figure S1), indicating that insertion of the SpdCas9 cassette did not disturb 2,3-BDO production. We next inserted the sgRNA-expressing cassette with spacers that target *fbp*, *pdh*, *ppc*, or *sps* and confirmed that separately knocking down these four genes enhanced 2,3-BDO production without apparent cell growth inhibition (Supplementary Figure S1).

To build the combinatorial CRISPRi library, for each gene, we designed sgRNAs with the spacers that have the highest on-target scores (sg1 or sg2, Figure 3C; Supplementary Table S1) for sequences located downstream the transcription start site (TSS) of *fbp*, *pdh*, *ppc*, or *sps*. We also designed a control sgRNA containing an identical handle region but with a sham spacer that targets no gene in cyanobacteria (sg0). Because gene suppression efficiency is inversely proportional to the distance from TSS (Hsu et al., 2020), sg0 would not repress gene expression; sg1 would confer weaker suppression, while sg2 would confer stronger repression. We labeled each sgRNA with a barcode (A: *fbp*; B: *pdh*; C: *ppc*; and D: *sps*; Supplementary Table S1) and constructed the combinatorial CRISPRi plasmid library (Figure 3D, Supplementary Figure S2). Each gene has three possible suppression levels (0: none; 1, weak; and 2: strong); thus, there are 3^4^ (=81) possible combinations. The combinatorial sgRNA library was integrated into the NSIII site of 7942-BDOdCas using SaCas9 to yield 7942-BDOdCas-library (Figure 3E). After antibiotic selection, 210 colonies were picked and cultured in shaker flasks. Almost all clones conferred higher 2,3-BDO titer than the parent 7942-BDOdCas strain, with an average titer of 1226.1 mg/L (Figure 3F).

### Effects of Combinatorial Gene Suppression on 2,3-BDO Production

**FIGURE 4:**
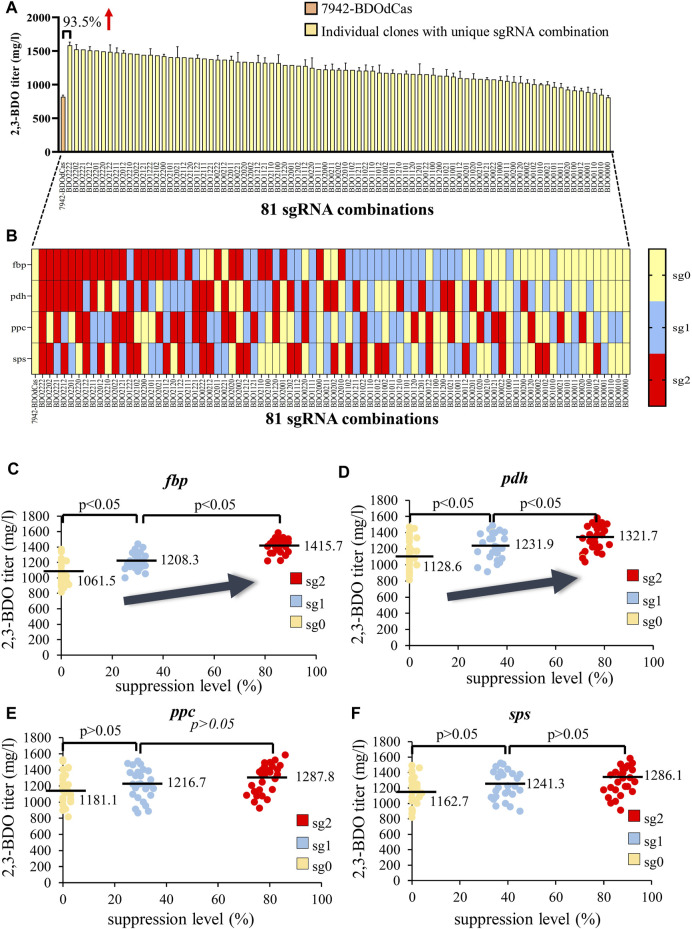
Effects of combinatorial gene suppression on 2,3-BDO production. **(A,B)** Correlation between the extracellular 2,3-BDO titer and the 81 sgRNA combinations (also see Supplementary Table S2). All the 210 clones were sequenced and designated based on the sgRNA combination. **(B)** Illustration of the targeting site (sg0, sg1, or sg2) at each gene (*fbp*, *pdh*, *ppc*, and *sps*) and the designated sgRNA combination. For instance, BDO2222 indicates that the sgRNA targeted four genes at sg2. BDO2202 indicates that the sgRNA targeted *fbp*, *pdh*, and *sps* at sg2 and *ppc* with sg0. **(C–F)** Effects of suppression of *fbp* **(C)**, *pdh* **(D)**, *ppc* **(E),** or *sps* **(F)** on the 2,3-BDO titer. The gene expression levels were measured by qRT-PCR, and suppression levels were calculated for each clone. The data represent the mean ± SD of at least three independent experiments and were statistically analyzed by one-way ANOVA. *p* < 0.05 was considered statistically significant.

With the barcode, we sequenced all the clones and verified the presence of 81 sgRNA combinations (Supplementary Table S2). Figures 4A,B illustrate the relationship between the extracellular 2,3-BDO titer and sgRNA targeting site. BDO2222 (inhibiting all four genes at sg2) conferred the highest titer (1583.8 mg/L), which was almost 2-fold that of the parent 7942-BDOdCas strain (818.4 mg/L). BDO2202 (sg2 for *fbp*, *pdh*, and *sps*; sg0 for *ppc*) yielded the second highest titer, while BDO0000 (inhibiting all four genes with sg0) did not improve 2,3-BDO synthesis.

To further verify the correlation between 2,3-BDO titer and individual gene suppression, we analyzed the gene suppression levels in each clone (Figures 4C–F). For each gene, sg0 conferred virtually no suppression (≈0–4%), while sg1 gave rise to mediocre suppression levels (≈25–45%), and sg2 conferred the strongest suppression (≈71–93%). For *fbp* (Figure 4C), suppression with sg1 significantly (*p* < 0.05) increased the average 2,3-BDO titer from 1061.5 mg/L (sg0) to ≈1208.3 mg/L (sg1). Suppression with sg2 further increased the average 2,3-BDO titer to 1415.7 mg/L. Likewise, we observed significantly higher 2,3-BDO titer when the *pdh* gene was suppressed at higher levels (Figure 4D). Repressing *ppc* and *sps* also increased the 2,3-BDO titer but without statistical significance (Figures 4E,F).

### Effects of Gene Suppression on Intracellular Metabolite Levels

**FIGURE 5:**
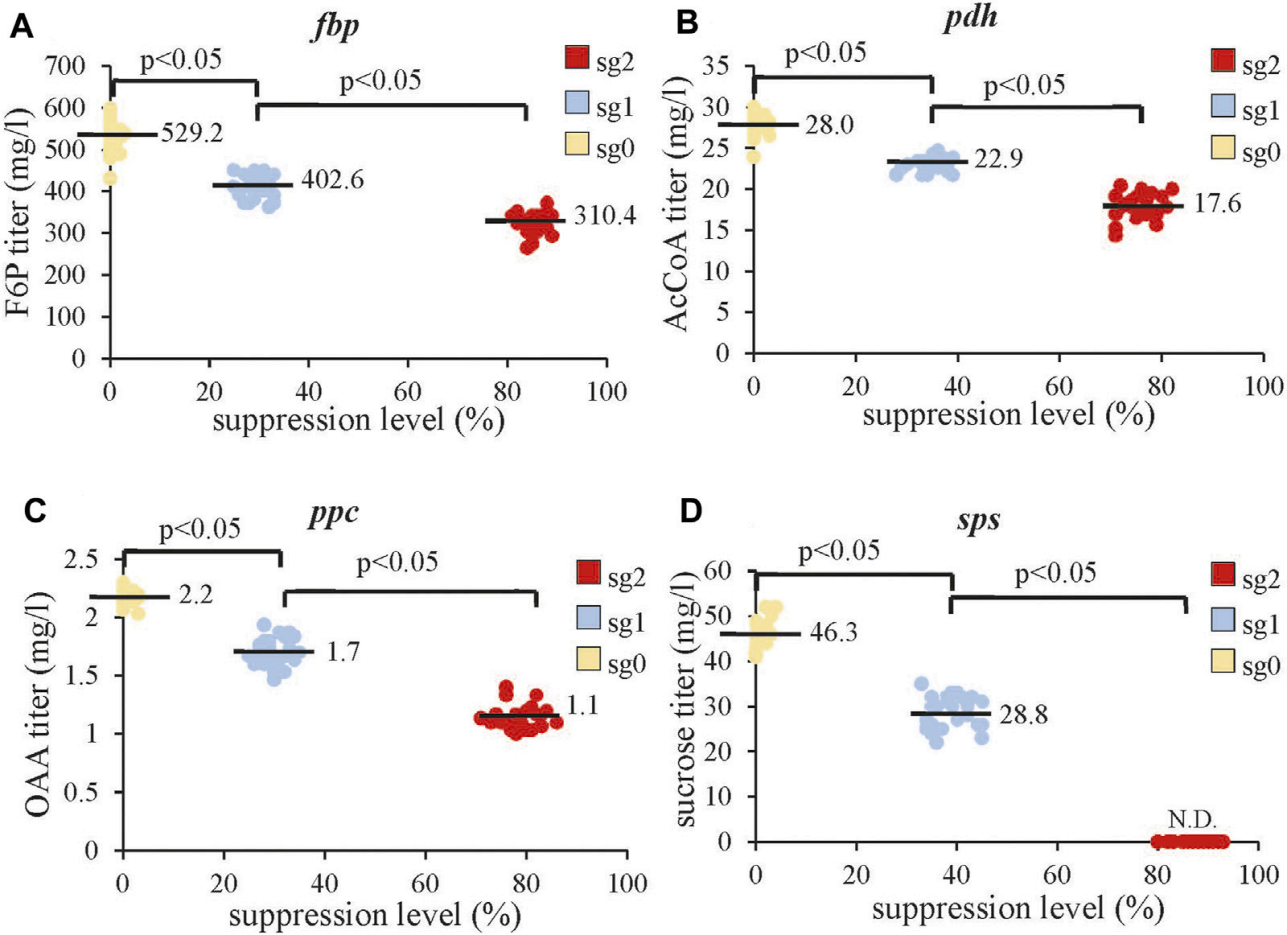
Effects of gene suppression on intracellular metabolite levels. **(A)** Effects of *fbp* suppression on F6P titer. **(B)** Effects of *pdh* suppression on the AcCoA titer. **(C)** Effects of *ppc* suppression on the OAA titer. **(D)** Effects of *sps* suppression on sucrose. The data represent the mean ± SD of at least three independent experiments and were statistically analyzed by one-way ANOVA. *p* < 0.05 was considered statistically significant. The intracellular F6P, AcCoA, OAA, and sucrose for all 210 clones were analyzed by HPLC-MS. N.D., not detectable.

Since the combinatorial CRISPRi library blocked different pathways, we also analyzed the concentrations of intracellular metabolites immediately downstream of the repressed gene in all the clones. We found that increasing the suppression of *fbp*, *pdh*, *ppc*, and *sps* concurrently decreased the titers of F6P, AcCoA, OAA, and sucrose, respectively, (Figures 5A–D). Of note, strong *sps* suppression at sg2 virtually abrogated sucrose production, suggesting complete blockade of this pathway.

### Effects of Multiple Genes and Two-Way Interactions

The aforementioned data revealed the effects of single genes on the responses (e.g. 2,3-BDO production). To gain more insight in the context of complex metabolic networks, we used response surface methodology to analyze how repression of multiple genes and their two-way interactions correlate with different responses (e.g. 2,3-BDO, F6P, AcCoA, OAA, and sucrose titers). We used a second-degree polynomial model (Table 1) for regression analysis of the experimental data from all 81 combinations as shown in Figures 4, 5. In this statistical model, Y_n_ indicates the response, X_n_ indicates the suppression level of each gene (*fbp*, *pdh*, *ppc*, or *sps*), X_m_X_n_ represents the two-way interactions of two genes, and C_n_ represents the effect coefficient.

**TABLE 1:** Effects of combinatorial gene suppression on 2,3-BDO production and target metabolite synthesis.

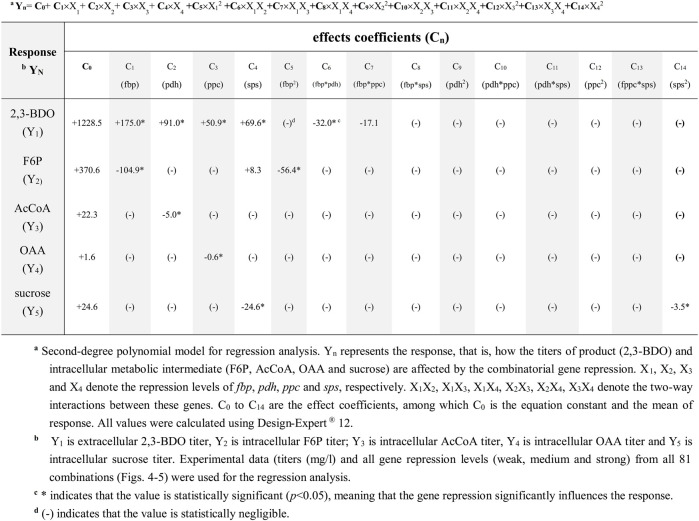															

For 2,3-BDO (Y_1_), the first four coefficients (C_1_, C_2_, C_3_, and C_4_) are positive and statistically significant (*, Supplementary Table S3), indicating that suppressing the genes (*fbp*, *pdh*, *ppc*, and *sps*) enhances the response (i.e. 2,3-BDO titer). The negative C_6_ (−32.0) and C_7_ (−17.1) suggests that simultaneously suppressing *fbp*/*pdh* (X_1_X_2_) and *fbp*/*ppc* (X_1_X_3_) decreases the 2,3-BDO titer. However, their values are tremendously lower than the constant C_0_ (+1228.5) and C_1_ (+175.0). Thus the two-way interactions are less significant. The values of other coefficients are lower than the threshold values and are negligible (-).

For F6P (Y_2_), C_1_ has a significantly higher negative value (-104.9) than C_2_, C_3_, and C_4_, indicating that suppressing *fbp* reduces the F6P titer but repressing *pdh*, *fbp*, and *ppc* does not. For AcCoA and OAA, C_2_ (−5.0) and C_3_ (−0.6) values are small because AcCoA and OAA titers are low (Figures 5B,C). Nonetheless, their values are statistically significant, indicating that suppressing *pdh* and *ppc* inhibits the production of AcCoA and OAA, respectively. Other coefficients are negligible, suggesting that two-way interactions barely influence the titers of AcCoA and OAA. For sucrose, C_4_ (−24.6) is statistically significant and virtually equal to C_0_ (+24.6), while other constants are mostly negligible. This echoes the finding that strong inhibition of *sps* completely suppresses sucrose production (Figure 5D). These data collectively indicated that suppressing *fbp*, *pdh*, *ppc*, and *sps* significantly (*p* < 0.05) reduce F6P, AcCoA, OAA, and sucrose synthesis, respectively, hence promoting the carbon flux rewiring toward 2,3-BDO synthesis. However, the levels of metabolites (F6P, AcCoA, OAA, and sucrose) are not markedly influenced by two-way interactions.

## Discussion

Despite the promise of photoautotrophic production of chemicals by cyanobacteria, the low volumetric product titer of 2,3-BDO (at levels of mg/l) impedes the application of cyanobacteria for industrial purposes and inspires the need to enhance the product titer (Shabestary et al., 2018). Although CRISPR and CRISPRi have emerged as promising tools to modulate the metabolic networks in cyanobacteria (Gordon et al., 2016; Huang et al., 2016; Li et al., 2016; Yao et al., 2016; Xiao et al., 2018), they are yet to be coupled to improve product synthesis in cyanobacteria.

Here, we first confirmed that SpCas9, SaCas9, and St1Cas9 can be used as orthogonal CRISPR systems for genome engineering of PCC7942 (Figure 1), and we utilized SaCas9 to integrate the synthetic pathway genes for 2,3-BDO synthesis (Figure 2). In addition to Cas9, in recent years, Cas12a has gained popularity as a genome editing tool. In contrast to Cas9, Cas12a only requires a single short crRNA to program target specificity and cleaves the DNA strands into staggered ends [instead of blunt ends cut by Cas9) (for review see (Hsu et al., 2019)]. Therefore, Cas12a may also be used in lieu of SaCas9 as an orthogonal system for integrating the CRISPRi system into the genome.

Despite the successful integration of the 2,3-BDO synthesis pathway, the 2,3-BDO titer was merely mediocre at 792.8 mg/L (Figure 2). Nonetheless, the orthogonality between SpCas9-and SaCas9-based CRISPR allows for integration of the SpdCas9-based CRISPRi system using SaCas9 to specifically knockdown endogenous genes and enhance 2,3-BDO production without overt cell growth inhibition (Supplementary Figure S1). Since CRISPRi can be used to fine-tune endogenous gene expression by the sgRNA target site design, such orthogonal CRISPR/Cas systems render simultaneous heterologous gene overexpression and endogenous gene knockdown in a multiplex and intricate fashion without mutual interference and may serve as a new toolbox for the combinatorial metabolic engineering of cyanobacteria.

To increase and redirect carbon flux toward 2,3-BDO production, glucose utilization and CO_2_ fixation in PCC7942 were recently enhanced together by engineering the glycolytic pathways and CBB cycle (Kanno et al., 2017). This study, similar to many other reports, attempts to improve bio-derived chemical synthesis (Wang et al., 2017; Liu et al., 2018; Yu et al., 2018; Cheah et al., 2020; Lee et al., 2020; Santos-Merino et al., 2021) and adopted a design-build-test-learn cycle by deleting and inserting genes step-by-step. However, this strategy is very time-consuming, and gene deletion often results in failure or slow cell growth during the strain improvement process (Kanno et al., 2017). This is not uncommon because deleting multiple genes often gives rise to unpredictable outcomes such as accumulation of toxic intermediates in the complex cellular environment (Zhao et al., 2021; Liu et al., 2022). For other products, several strategies, such as reducing the loss of intermediates to competing pathways (Liu et al., 2022), genome-scale modeling (Broddrick et al., 2016), and modular engineering (Liu et al., 2019), have been used to assess or tune cellular metabolism. Moreover, metabolomic techniques such as mass spectrometry (MS) and nuclear magnetic resonance (NMR) are used to identify key metabolites that contribute to product synthesis (Kato et al., 2022). For instance, isotopically nonstationary metabolic flux analysis (INST-MFA) is used to identify gene targets for increasing aldehyde production in PCC7942 (Cheah et al., 2020). Conversely, pooled CRISPRi screening is applied to mammalian cells (Du et al., 2017; Liu et al., 2017) and bacteria (Wang et al., 2018; Peters et al., 2019) to screen gene essentiality and diverse phenotypes. Recently, the pooled CRISPRi strategy was used in cyanobacterium PCC6803 to screen genes essential for cell growth (Yao et al., 2020). However, this methodology is yet to be combined with the orthogonal CRISPR system to enhance bio-derived chemical production in PCC7942.

In contrast to the aforementioned strategies and owing to the ability of orthogonal SaCas9 to integrate the SpdCas9-based CRISPRi library for multiplex suppression, we developed a combinatorial CRISPRi library approach for rapid screening and improvement of 2,3-BDO production in PCC7942. This methodology allowed us to inhibit *fbp*, *pdh*, *ppc*, and *sps* at different levels in a combinatorial way to enhance 2,3-BDO production (Figure 3) and confirm that differentially inhibiting all the four genes enhances the 2,3-BDO titer to varying degrees (Figure 4). Particularly, repression of all the four genes at the strongest levels imparted additive effects to improve 2,3-BDO titers by 93.5%–1583.8 mg/L (Figures 4A,B).

Furthermore, we combined the CRISPRi library data and regression analysis to quantify the influences of each gene and gene pairs on the titers of desired 2,3-BDO and undesired intracellular metabolites (Figures 4, 5; Table 1). In addition to verifying that simultaneously inhibiting all the four genes (*fbp*, *pdh*, *ppc*, and *sps*) enhances the 2,3-BDO titer and decreases the respective titers of metabolites downstream of the gene, we found more significant effects of *fbp* and *pdh* suppression on 2,3-BDO production as their effect coefficients are larger than those of *ppc* and *sps* (Table 1). These effects may be attributed to the roles of these genes on carbon flux partition. *fbp* regulates F6P synthesis in the CBB cycle, which can be converted to sucrose (Figure 3A) or glycogen (Hickman et al., 2013) as storage compounds. Since the carbon for 2,3-BDO synthesis can be provided directly by G3P without F6P (Figure 3A), inhibiting *fbp* reduces unnecessary F6P formation without significant impairment of 2,3-BDO synthesis, thus exerting profound effects on 2,3-BDO titer increase. Conversely, PYR is an important branching point for cyanobacterial production of 2,3-BDO (Oliver et al., 2013; Savakis et al., 2013; Oliver et al., 2014); thus, increasing the intracellular PYR pool and redirecting the carbon flux toward 2,3-BDO production is critical. *pdh* is responsible for converting PYR to AcCoA to enter TCA cycle or produce other byproducts such as acetate (Hirokawa et al., 2020); thus, inhibiting *pdh* markedly blocks the carbon flux toward AcCoA, TCA cycle, and other byproducts, hence augmenting 2,3-BDO production. Our finding concurs with the recent reports that overexpressing *pdh* increases the carbon partition into AcCoA and acetate/isopropanol production (Hirokawa et al., 2020), while *pdh* knockdown through antisense RNA elevates PYR partition (Cheah et al., 2020).

In contrast, inhibiting *sps* only suppresses sucrose synthesis, hence imparting weaker effects. In addition, *ppc* is helpful to OAA replenishment and *ppc* inhibition, indeed, improves 2,3-BDO formation. However, OAA can be recycled by TCA cycle itself, which might offset the effect of inhibiting the *ppc* gene and result in less significant effect on 2,3 BDO production. We also unveiled that the two-way interactions between *fbp*/*pdh* and *fbp*/*ppc* slightly decreases the 2,3-BDO titer, but their effects are overshadowed by suppressing individual genes (Table 1). By and large, the effect of two-way interactions between gene pairs on metabolite titers are less significant than that of single genes (Table 1), indicating that these four genes can be repressed simultaneously to enhance 2,3-BDO production without mutual interference.

In summary, we identified the orthogonal CRISPR/Cas9 systems for the integration of synthetic pathway genes and the CRISPRi system to regulate the gene expression/suppression levels in PCC7942 to intricately modulate the metabolic flux and product synthesis. We also developed a combinatorial CRISPRi library strategy that allows for systematic and rational analysis to elucidate the effects of individual genes and gene pairs on 2,3-BDO production. The CRISPRi library approach enables us to yield a strain that improves 2,3-BDO production by 93.5% and is less time-consuming and laborious than traditional approaches. Since re-engineering glucose utilization and reinforcing CO_2_ assimilation substantially increase 2,3-BDO synthesis (Kanno et al., 2017), our approach may be exploited to investigate the roles of genes in glycolytic pathways and CBB cycle to further increase and redirect the carbon flux to 2,3-BDO production.

Although fast-growing cyanobacteria strains, such as UTEX 2973 (Yu et al., 2015), have been identified, their genetic backgrounds remain poorly understood. Nonetheless, our CRISPRi library strategy may be extrapolated to unravel genes critical for bioproduct synthesis in these fast-growing strains. Furthermore, our combinatorial CRISPRi library may be combined with metabolomics (Kato et al., 2022) to unveil the roles of genes essential for production of other chemical products and accelerate the application of cyanobacteria for biosynthesis.

## Materials and Methods

### Culture and Transformation of *S. elongatus* PCC7942

PCC7942 cells were cultured using BG-11 medium in a 30 °C incubator (600SR, Hipoint), with illumination from continuous cool white fluorescent light (intensity≈70 μmol//m^2^⋅s). For suspension culture, PCC7942 cells were cultivated using 40 ml medium in a 250-ml shaker flask (gyratory shaking at 100 rpm) until OD_730_ reached ≈0.6 (for transformation) or until 11 days (for 2,3-BDO production). For plate culture, PCC7942 cells were streaked onto 90-mm plates containing 40 cm^3^ BG-11/agar medium supplemented with 1 mM sodium thiosulfate and cultured for 7–9 days until the colonies developed. Plasmid transformation into cells was performed as described previously (Li et al., 2016).

### Construction of Plasmids and Engineered 7,942 Strains

All the cloning protocols were performed with standard methods using *E. coli* DH5α strain as the host. pSpCas9, pSaCas9, and pStCas9 that expressed SpCas9, SaCa9, and St1Cas9, respectively, were constructed previously (Sung et al., 2019). For sgRNA expression, we used psgLacZ-Sp, psgLacZ-Sa, and psgLacZ-St (Sung et al., 2019) as the backbone but swapped the spacer sequence to target the NSI site (yielding psgRNA-NSI-Sp, psgRNA-NSI-Sa, or psgRNA-NSI-St) or the NSII site (yielding psgRNA-NSII-Sp or psgRNA-NSII-Sa).

The plasmids for integration into the three neutral sites (NSI, NSII, or NSIII) were constructed based on pSyn-1, pSyn-2, or pSyn-3 (Invitrogen). These plasmids contained the spectinomycin resistance gene (Spc^R^), a multiple cloning site (MCS), downstream of sc promoter, a rrnB transcription terminator, and flanking sequences homologous to the NSI, NSII, or NSIII site. We PCR-amplified the inducible smt promoter and subcloned it into pSyn-1 to replace the sc promoter and yielded psmt-NSI. For pNSII, we replaced Spc^R^ and sc promoter by the kanamycin resistance gene (Km^R^) and LacO1 promoter to yield pLacO1-NSII. For NSIII, Spc^R^ was replaced by the chloramphenicol resistance gene (Cm^R^) to yield pCm-NSIII.

To clone the synthetic 2,3-BDO pathway, we chemically synthesized *alsS* from *Bacillus subtilis*, *alsD* from *Enterobacter cloacae*, and *adh* from *Clostridium beijerinckii* (Genomics BioSci & Tech, Taiwan) with flanking *Avr*II and *Xho*I. The three genes were subcloned into the MCS of pLacO1-NSII to yield pNSII-23BDO. We transformed the wild-type PCC7942 strain with only 2000 ng of pNSII-23BDO as the control group. In parallel, we co-transformed 2000 ng of pNSII-23BDO with 500 ng of pSpCas9 and 500 ng of pSgRNA-NSII-Sp (or 2000 ng of pNSII-23BDO with 500 ng of pSaCas9 and 500 ng of pSgRNA-NSII-Sa). The cells were streaked to plates containing kanamycin (Km), and colonies were picked and segregated as described (Li et al., 2016). The colony numbers were counted using a colony counter, and colony-forming units (CFUs) were obtained. After segregation, five colonies were picked for PCR analyses using two primer pairs targeting the left and right junctions at the integration site. The colonies with correct integration without contaminating bands were subcultured, stored at -80 °C, and designated as the 7942-BDO strain.

### Construction of CRISPRi Library and Engineered 7,942 Strain

To build the combinatorial CRISPRi library, we first PCR-amplified *SpdCas9* together with the trc promoter from pBac-SpdCas9 (Hsu et al., 2020). The PCR amplicon was subcloned into psmt-NSI to yield pNSI-dCas9. pNSI-dCas9 (2000 ng) was transformed into the 7942-BDO strain, and *SpdCas9* integration into the NSI site was verified as described previously. The resultant strain was subcultured, stored at -80 °C, and designated as 7942-BDOdCas.

For the sgRNA library, we chose to inhibit four genes (*fbp*, *pdh*, *ppc*, and *sps*) and calculated the on-target scores (Supplementary Table S1) using online software benchling (https://www.benchling.com/). We designed and chemically synthesized the partially overlapping oligonucleotides 1 to 8 to encode the sgRNA cassette, unique barcode, and Cm^R^ (Supplementary Figure S2). The sequences for oligonucleotides 3 and 4 were designed to encode a non-targeting spacer (sg0) or the spacer that has the highest on-target score (sg1 or sg2). Oligonucleotide 8 was designed to encode the unique barcode (Supplementary Table S1). The sequences for oligonucleotides 3, 4, and 8 are different for different gene targets. Using these oligonucleotides for gene assembly, we synthesized DNA amplicons encoding sgRNA cassettes with unique barcodes and Cm^R^ by overlapping PCR. These sgRNA target sg0, sg1, and sg2 for *fbp* (A), *pdh* (B), *ppc* (C), and *sps* (D) as shown in Supplementary Table S1. As shown in Supplementary Figure S2, the three DNA amplicons for the *fbp* sgRNA (A) were cloned into pCm-NSIII to yield three different psgRNA(A): psgRNA (A2), psgRNA (A1), and psgRNA (A0). In step 2, we cloned the three DNA amplicons for *pdh* (B) into three psgRNA(A) to yield nine different psgRNA (AB). In step 3, we cloned the three DNA amplicons for *ppc* (C) into nine different psgRNA (AB) to yield 27 unique psgRNA (ABC). In step 4, we cloned the three DNA amplicons for *sps* (D) into the 27 different psgRNA (ABC) to yield 81 different psgRNA (ABCD). The 81 different plasmids were verified using primers P1 and P2 (Supplementary Figure S2), owing to the unique barcode sequences. The 81 plasmids were pooled and transformed into 7942-BDOdCas for integration into the NSIII site using the SaCas9 system as described previously to yield the 7942-BDOdCas-library. The cells were spread to plates, and colonies were picked for analyses.

### Cell Death Analysis

To analyze cell death, we transformed PCC7942 cells with the Cas9-expressing plasmid (e.g., pSpCas9, pSaCas9, or pStCas9) at different doses (1,000, 2000, or 4,000 ng). Alternatively, we co-transformed the cells with equal amounts of Cas9-expressing and sgRNA-expressing plasmids (e.g., pSpCas9/psgRNA-NSI-Sp or pSpCas9/psgRNA-NSI-Sp). The transformed cells were diluted 10^6^-fold and streaked onto the BG-11/agar plate. The nontransformed cells were similarly diluted and streaked as the control. After 7–9 days, the colony numbers were counted and CFUs were calculated. The survival rate was calculated as CFU/CFU_control_×100%. The death rate was calculated as (1-CFU/CFU_control_)×100%.

### 2,3-BDO Analysis and Intracellular Metabolite Analyses

The extracellular 2,3-BDO titer was analyzed by GC-BID (Shimatsu, Japan) using the TG-WAXMS column (Shimatsu). The cells were cultured in 40 ml medium in the shaker flask for 11 days. After harvesting and centrifugation (6,000×g, 15 min), the supernatant was diluted 10-fold and analyzed using GC-BID. Meanwhile, 2,3-BDO standard (Sigma) was diluted to 200, 100, 50, 25, and 10 mg/L and analyzed similarly to establish the standard curve. The 2,3-BDO titers were determined based on the area of the signal and the standard curve.

For the analysis of intracellular metabolites F6P, AcCoA, OAA, and sucrose, the cells were cultured in 40 ml BG-11 medium for 11 days and 1 ml of the supernatant was withdrawn. After centrifugation, the cells were lysed using 100 μL TE buffer containing 2 mg/ml lysozyme in a 37 °C water bath for 20 min, followed by centrifugation (12,000×g, 5 min). Subsequently, 500 μL supernatant was analyzed by HPLC-MS (Shimadzu) using Hypersil™ BDS C18 HPLC Column (5 μm, 10 × 250 mm, Thermo Fisher) and 5% acetonitrile as the mobile phase. The standard F6P, AcCoA, OAA, and sucrose (Sigma) were diluted to 1,000, 500, 100, 10, and 1 mg/L and analyzed similarly to generate the standard curve.

### Quantitative Real-Time Reverse Transcription PCR

For gene expression, a single clone was picked and subcultured in 40 ml medium in a 250-ml shaker flask for 11 days, followed by centrifugation and lysis with the lysozyme as described previously. Approximately 3–5 μg total RNA was extracted using TRIzol^®^ and mixed with SYBR^®^ Green Master Mix and gene-specific primers. The mixture was subjected to real-time PCR as described (Pham et al., 2020). The gene expression levels were calculated by software (LightCycler^®^ 96 SW 1.1, Roche) using the *glgc* gene as the internal control. The data were normalized with the expression levels in the 7942-BDOdCas strain as the reference. The suppression levels were calculated as (1-expression level)×100%.

### Statistical Analysis

All the quantitative data represent the mean ± SD of at least three independent culture experiments and were analyzed using Student’s *t*-test or one-way ANOVA. *p* < 0.05 was considered significant. The CRISPRi library data, including gene suppression levels, titers of 2,3-BDO, F6P, AcCoA, OAA, and sucrose, were analyzed by the statistical software Design-Expert^®^ 12 using the response surface methodology as the statistical model. We used a second-order polynomial equation for regression analyses of how inhibition of multiple genes and their two-way interactions correlate with different responses (e.g., 2,3-BDO, F6P, AcCoA, OAA, and sucrose titers) from the experimental data of all 81 combinations of the CRISPRi library (See Results).

## Data Availability

The original contributions presented in the study are included in the article/Supplementary Material. Further inquiries can be directed to the corresponding author.
