# Supplementary material for: Combinatorial CRISPR Interference Library for Enhancing 2,3-BDO Production and Elucidating Key Genes in Cyanobacteria
Source: Front Bioeng Biotechnol. 2022 Jun 21;10:913820. doi: 10.3389/fbioe.2022.913820 (PMC9253771; doi:10.3389/fbioe.2022.913820)
Supplement: Supplementary file 1 [file DataSheet1.PDF]

Combinatorial CRISPR Interference Library for Enhancing 2,3-BDO Production and Elucidating Key Genes in Cyanobacteria

Hung Li[1], Nam Ngoc Pham[1], Claire R. Shen[1], Chin-Wei Chang[1], Yi Tu[2], Yi-Hao Chang[1], Jui Tu[3], Mai Thanh Thi Nguyen[4] and Yu-Chen Hu[1, 5*]

[1]Department of Chemical Engineering, National Tsing Hua University, Hsinchu, Taiwan
[2]Department of Life Science, National Taiwan University, Taipei, Taiwan
[3]Department of Chemical Engineering, National Taiwan University, Taipei, Taiwan
[4]Faculty of Chemistry, University of Science, Vietnam National University Ho Chi Minh City, Ho Chi Minh City, Vietnam
[5]Frontier Research Center on Fundamental and Applied Sciences of Matters, National Tsing Hua University, Hsinchu, Taiwan.

Running Title: CRISPRi library to improve 2,3-BDO production and decipher key genes

*Corresponding Author
Phone: (886)3-571-8245
FAX: (886)3-571-5408
Email: yuchen@che.nthu.edu.tw
ORCID: https://orcid.org/0000-0002-9997-4467

Supplementary Figures

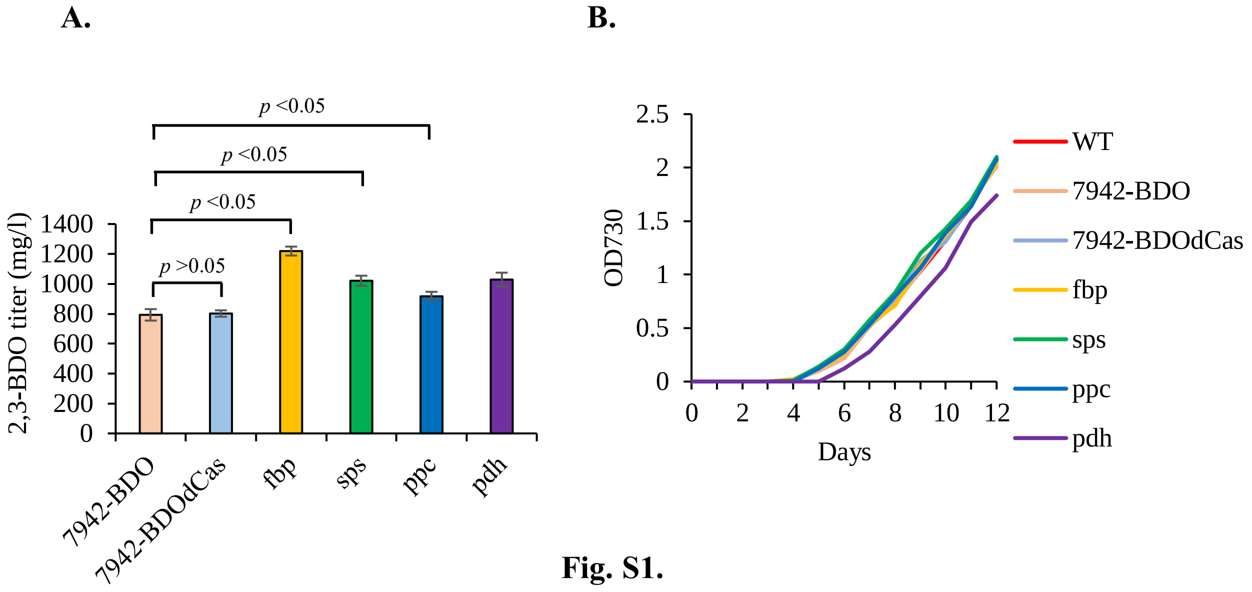

Fig. S1.

Fig S1. Effects of gene suppression 2,3-BDO titers and PCC7942 growth. (A) 2,3-BDO titer. (B) Cell growth. We integrated synthetic 2,3-BDO pathway genes into NSII site of PCC7942 using SaCas9 system to yield 7942-BDO strain. We next integrated the SpdCas9 expression cassette into NSI site of 7942-BDO using the SaCas9 system and yielded 7942-BDOdCas strain. We next constructed 4 plasmids which harbored NSIII homology arms and sgRNA-expressing cassette with spacers that target the protospacer downstream the transcription start site (TSS) of *fbp*, *pdh*, *ppc* or *sps* (sg2, Fig. 3C). We separately transformed these plasmids into 7942-BDOdCas strain. These engineered strains and control cells were cultured in shake flasks for 11 days and monitored for 2,3-BDO titer by GC. Cell growth was measured by OD_{730} using a plate reader. WT, wild-type. 7942-BDOdCas produced similar levels of BDO (818.4 mg/l) as compared with 7942-BDO (792.8 mg/l), indicating that insertion of SpdCas9 cassette into NSI site did not disturb 2,3-BDO production. These data also confirmed that separately knocking down these 4 genes enhanced 2,3-BDO production without apparent cell growth inhibition.

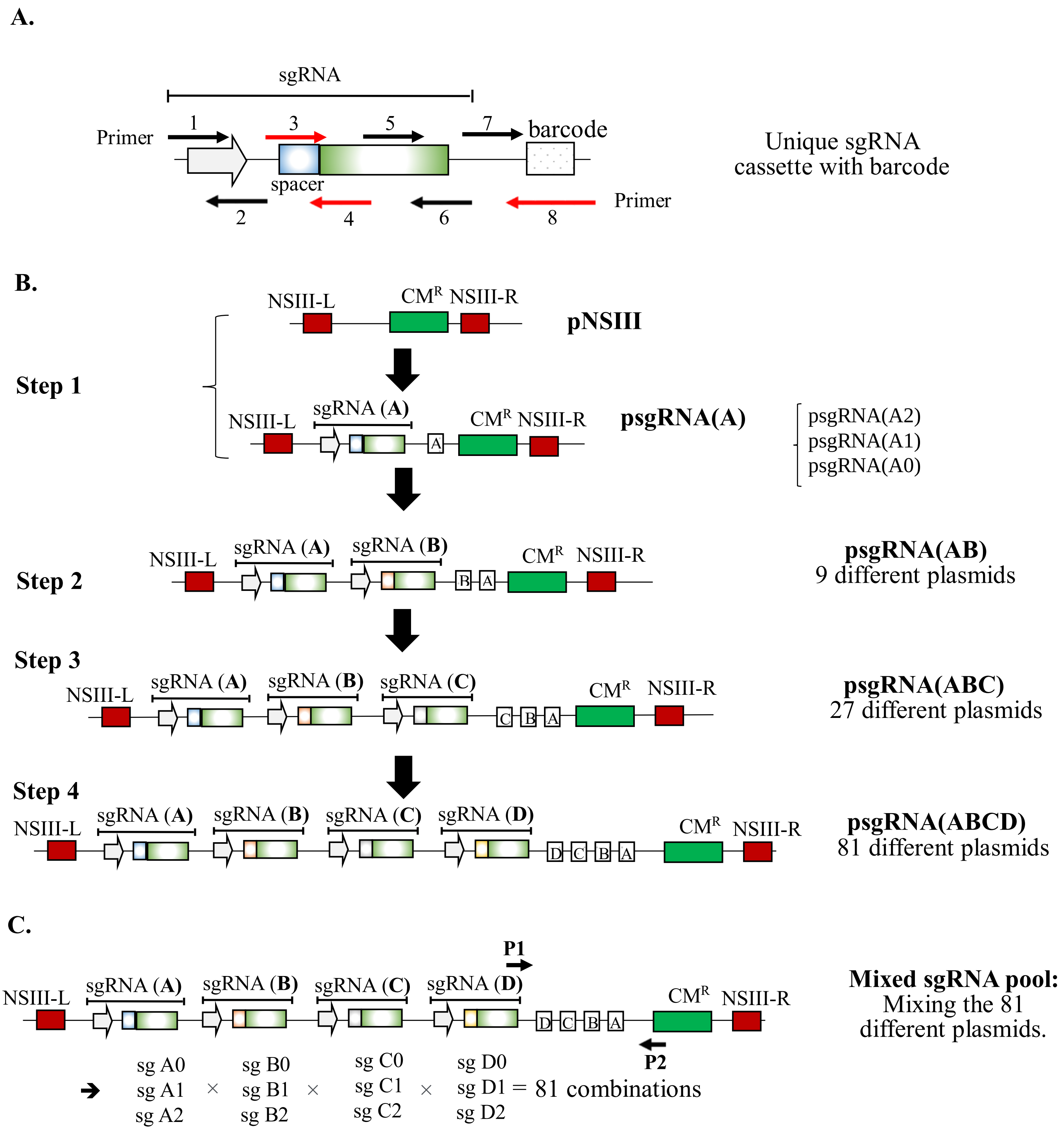

Fig. S2. Construction of CRISPRi library. (A) Design of unique sgRNA. (B) Process to build the CRISPRi library. (C) Illustration of the 81 combinations in the CRISPRi library. We first designed and synthesized partially overlapping oligonucleotides 1 to 8 to encode the sequences for the sgRNA cassette and unique barcode. The sequences for oligonucleotides 3 and 4 were designed to encode a non-targeting spacer (sg0) or the spacer that has the highest on-target score (sg1 and sg2,

Fig. 3C and Table S1). Oligonucleotide 8 was designed to encode the unique barcode. The sequences for oligonucleotides 3, 4 and 8 are different for different gene targets. Using these oligonucleotides for gene assembly, we synthesized DNA amplicons encoding sgRNA cassettes with unique barcodes by overlap PCR. These sgRNA target sg0, sg1 and sg2 for *fbp* (A), *pdh* (B), *ppc* (C), *sps* (D) (Table S1). As shown in Step 1, the 3 DNA amplicons for *fbp* sgRNA (A) were cloned into pNSIII to yield 3 different psgRNA(A): psgRNA(A2), psgRNA(A1) and psgRNA(A0). In Step 2, we cloned the 3 DNA amplicons for *pdh* (B) into 3 psgRNA(A) to yield 9 different psgRNA(AB). In Step 3, we cloned the 3 DNA amplicons for *ppc* (C) into 9 different psgRNA(AB) to yield 27 unique psgRNA(ABC). In Step 4, we cloned the 3 DNA amplicons for *sps* (D) into the 27 different psgRNA(ABC) to yield 81 different psgRNA(ABCD). The 81 different plasmids were verified using primers P1 and P2 thanks to the unique barcode sequences. The 81 plasmids were pooled and transformed into 7942-BDOdCas for integration into NSIII site to yield the 7942-BDOdCas-library. Cm^R, chloramphenicol resistance gene.

Supplementary Tables

Table S1. sgRNA and barcode design

Gene	Name	Location relative to TSS[a]	PAM	Spacer sequence	[b]On-target score	Barcode
**Ø**	Sg0	none	-	ATTAATTGTCAATTCGAA	-	CCAGAATA
fbp	sg2	+101	GGG	AAGAAGGCTGTCGATTCTCG	64.0	GATTGTAC
	sg1	+582	CGG	GTCGATGTCGATCTCAACGT	74.1	GATCCATA
sps	sg2	+110	CGG	GGCCCATAATCCTTGGTGAG	63.6	ATAAACGC
	sg1	+1114	TGG	CCGCCAAGACATCAACCAGA	60.7	ATAATATG
ppc	sg2	+56	TGG	GTTCTGTGTCCAGTCAGACG	71.4	TTCAGGCA
	sg1	+1019	TGG	CCCCTGCCCGAATATCGGGA	61.5	TTCGTCCA
pdh	sg2	+190	TGG	TGCCGCCTTCGAGGAACCCA	55.7	TCGTTAAC
	sg1	+622	TGG	AGTGCCCGGCCTAAAGATTG	60.2	TCGGATTA

[a]TSS: transcription start site

[b]On-target scores were calculated by online software benchling (https://www.benchling.com/).

Table S2. 81 sgRNA combinations, clone names and average 2,3-BDO titer. The 4 numbers in the clone names indicate the suppression strength of the 4 genes: *fbp*, *pdh*, *ppc*, *sps*. 0 denotes no repression; 1 indicates weak suppression; 2 indicates strong repression.

Clone name	sgRNA combination	Colony No.	Avg. titer (mg/l)
**BDO0000**	fbp0, pdh0, ppc0, sps0	1,4,11,73,150	808.3
**BDO0001**	fbp0, pdh0, ppc0, sps1	19,78, 145	889.6
**BDO0002**	fbp0, pdh0, ppc0, sps2	34,136,169	1025.1
**BDO0010**	fbp0, pdh0, ppc1, sps0	3,71	848.6
**BDO0011**	fbp0, pdh0, ppc1, sps1	9,17,25,107,165,199	956.7
**BDO0012**	fbp0, pdh0, ppc1, sps2	83,120	911.6
**BDO0020**	fbp0, pdh0, ppc2, sps0	21,170	926.5
**BDO0021**	fbp0, pdh0, ppc2, sps1	88,134,196	1000.7
**BDO0022**	fbp0, pdh0, ppc2, sps2	16,208	1076.7
**BDO0100**	fbp0, pdh1, ppc0, sps0	63,103,151	914.8
**BDO0101**	fbp0, pdh1, ppc0, sps1	98,149	965.7
**BDO0102**	fbp0, pdh1, ppc0, sps2	33,52,127	1005.3
**BDO0110**	fbp0, pdh1, ppc1, sps0	106.173	877.4
**BDO0111**	fbp0, pdh1, ppc1, sps1	74,161	1054.9
**BDO0112**	fbp0, pdh1, ppc1, sps2	27,49,168,203	1099.2
**BDO0120**	fbp0, pdh1, ppc2, sps0	113,146	1029.1
**BDO0121**	fbp0, pdh1, ppc2, sps1	156,178,207	1077.2
**BDO0122**	fbp0, pdh1, ppc2, sps2	72	1153.6
**BDO0200**	fbp0, pdh2, ppc0, sps0	80,128	1039.2
**BDO0201**	fbp0, pdh2, ppc0, sps1	137,204	1093.0
**BDO0202**	fbp0, pdh2, ppc0, sps2	126,147	1220.6
**BDO0210**	fbp0, pdh2, ppc1, sps0	160	1083.8
**BDO0211**	fbp0, pdh2, ppc1, sps1	35,65,102	1222.7
**BDO0212**	fbp0, pdh2, ppc1, sps2	154	1371.1
**BDO0220**	fbp0, pdh2, ppc2, sps0	53,135	1249.9
**BDO0221**	fbp0, pdh2, ppc2, sps1	37,162	1341.5
**BDO0222**	fbp0, pdh2, ppc2, sps2	112,129	1371.5
**BDO1000**	fbp1, pdh0, ppc0, sps0	12,28,94	1065.2
**BDO1001**	fbp1, pdh0, ppc0, sps1	58,89,133	1118.7
**BDO1002**	fbp1, pdh0, ppc0, sps2	140	1174.0

**BDO1010**	fbp1, pdh0, ppc1, sps0	68,104,201	1001.9
**BDO1011**	fbp1, pdh0, ppc1, sps1	79,116	1170.8
**BDO1012**	fbp1, pdh0, ppc1, sps2	18,55,69	1176.9
**BDO1020**	fbp1, pdh0, ppc2, sps0	82,124,176,193	1088.2
**BDO1021**	fbp1, pdh0, ppc2, sps1	23,66,114	1130.4
**BDO1022**	fbp1, pdh0, ppc2, sps2	117,155	1207.4
**BDO1100**	fbp1, pdh1, ppc0, sps0	14,172	1145.9
**BDO1101**	fbp1, pdh1, ppc0, sps1	40,101	1163.1
**BDO1102**	fbp1, pdh1, ppc0, sps2	111	1219.2
**BDO1110**	fbp1, pdh1, ppc1, sps0	50,73	1206.1
**BDO1111**	fbp1, pdh1, ppc1, sps1	99	1229.9
**BDO1112**	fbp1, pdh1, ppc1, sps2	42,122,141,205	1277.4
**BDO1120**	fbp1, pdh1, ppc2, sps0	90,192	1158.4
**BDO1121**	fbp1, pdh1, ppc2, sps1	166,179	1324.7
**BDO1122**	fbp1, pdh1, ppc2, sps2	118,148	1396.6
**BDO1200**	fbp1, pdh2, ppc0, sps0	48	1131.8
**BDO1201**	fbp1, pdh2, ppc0, sps1	110,159	1154.7
**BDO1202**	fbp1, pdh2, ppc0, sps2	130,200	1281.4
**BDO1210**	fbp1, pdh2, ppc1, sps0	175,197	1166.3
**BDO1211**	fbp1, pdh2, ppc1, sps1	13,85,139,142	1208.0
**BDO1212**	fbp1, pdh2, ppc1, sps2	29,91,171	1331.0
**BDO1220**	fbp1, pdh2, ppc2, sps0	163	1291.3
**BDO1221**	fbp1, pdh2, ppc2, sps1	100,209	1377.0
**BDO1222**	fbp1, pdh2, ppc2, sps2	15,81,97	1442.5
**BDO2000**	fbp2, pdh0, ppc0, sps0	20,30,56	1225.6
**BDO2001**	fbp2, pdh0, ppc0, sps0	180	1290.4
**BDO2002**	fbp2, pdh0, ppc0, sps2	59,191	1333.6
**BDO2010**	fbp2, pdh0, ppc1, sps0	92,158,174	1219.4
**BDO2011**	fbp2, pdh0, ppc1, sps1	109,164,167	1368.1
**BDO2012**	fbp2, pdh0, ppc1, sps2	95,152	1472.1
**BDO2020**	fbp2, pdh0, ppc2, sps0	153	1338.9
**BDO2021**	fbp2, pdh0, ppc2, sps1	115,138	1406.3
**BDO2022**	fbp2, pdh0, ppc2, sps2	39,194	1456.8
**BDO2100**	fbp2, pdh1, ppc0, sps0	5,22,77	1321.6
**BDO2101**	fbp2, pdh1, ppc0, sps1	144,206	1408.3
**BDO2102**	fbp2, pdh1, ppc0, sps2	108	1433.8
**BDO2110**	fbp2, pdh1, ppc1, sps0	121,198	1323.6
**BDO2111**	fbp2, pdh1, ppc1, sps1	45	1387.0

**BDO2112**	fbp2, pdh1, ppc1, sps2	122	1405.9
**BDO2120**	fbp2, pdh1, ppc2, sps0	84,202	1399.5
**BDO2121**	fbp2, pdh1, ppc2, sps1	42	1442.9
**BDO2122**	fbp2, pdh1, ppc2, sps2	24,62,132,210	1486.9
**BDO2200**	fbp2, pdh2, ppc0, sps0	87,157	1422.0
**BDO2201**	fbp2, pdh2, ppc0, sps1	93	1508.0
**BDO2202**	fbp2, pdh2, ppc0, sps2	86,143	1524.8
**BDO2210**	fbp2, pdh2, ppc1, sps0	105	1462.7
**BDO2211**	fbp2, pdh2, ppc1, sps1	46,177	1478.3
**BDO2212**	fbp2, pdh2, ppc1, sps2	26,132	1508.4
**BDO2220**	fbp2, pdh2, ppc2, sps0	109	1493.5
**BDO2221**	fbp2, pdh2, ppc2, sps1	70,195	1522.5
**BDO2222**	fbp2, pdh2, ppc2, sps2	76,96	1583.8

Table S3. Statistical values of factors with significance. Only the parameters with p values less than 0.05 are shown. Significant p values indicate that suppression of the gene (or gene pair) affect the response.

**Response Y_1: 2,3-BDO**		
Source	P value	Significant
fbp	< 0.0001	+
pdh	< 0.0001	+
ppc	< 0.0001	+
sps	< 0.0001	+
fbp pdh*	0.0004	+

**RESPONSE Y_2: F6P**		
Source	P value	Significant
fbp	< 0.0001	+
sps	0.0420	+
fbp^2	< 0.0001	+

**RESPONSE Y_3: ACCOA**		
Source	P value	Significant
pdh	< 0.0001	+

**RESPONSE Y_4: OAA**		
Source	p-value	Significant
ppc	< 0.0001	+

**RESPONSE Y_5: SUCROSE**		
Source	P value	Significant
sps	< 0.0001	+
sps^2	< 0.0001	+
